# Role of cardiovascular magnetic resonance in acute and chronic ischemic heart disease

**DOI:** 10.1007/s10554-017-1116-0

**Published:** 2017-03-18

**Authors:** A. Baritussio, A. Scatteia, C. Bucciarelli-Ducci

**Affiliations:** Bristol Heart Institute, Bristol NIHR Cardiovascular Biomedical Research Unit (BRU), Upper Maudlin Street, Bristol, BS2 8HW UK

**Keywords:** Cardiovascular magnetic resonance, Acute myocardial infarction, Chronic ischemic heart disease

## Abstract

Cardiovascular magnetic resonance (CMR) is a multi-parametric, multi-planar, non-invasive imaging technique, which allows accurate determination of biventricular function and precise myocardial tissue characterization in a one-stop-shop technique, free from the use of ionizing radiations. Though CMR has been increasingly applied over the last two decades in every-day clinical practice, its widest application has been in the assessment of ischemic cardiomyopathy.

## Acute myocardial infarction

Coronary artery disease (CAD) is the leading cause of death worldwide [1]. Although the incidence rate of ST-elevation myocardial infarction (STEMI) has declined, that of non-ST elevation myocardial infarction (NSTEMI) has increased over the last few decades [1]. Diagnosis usually relies on clinical history and electrocardiographic changes, while imaging, mainly trans-thoracic echocardiography (TTE), is usually deferred.

**Fig. 1:**
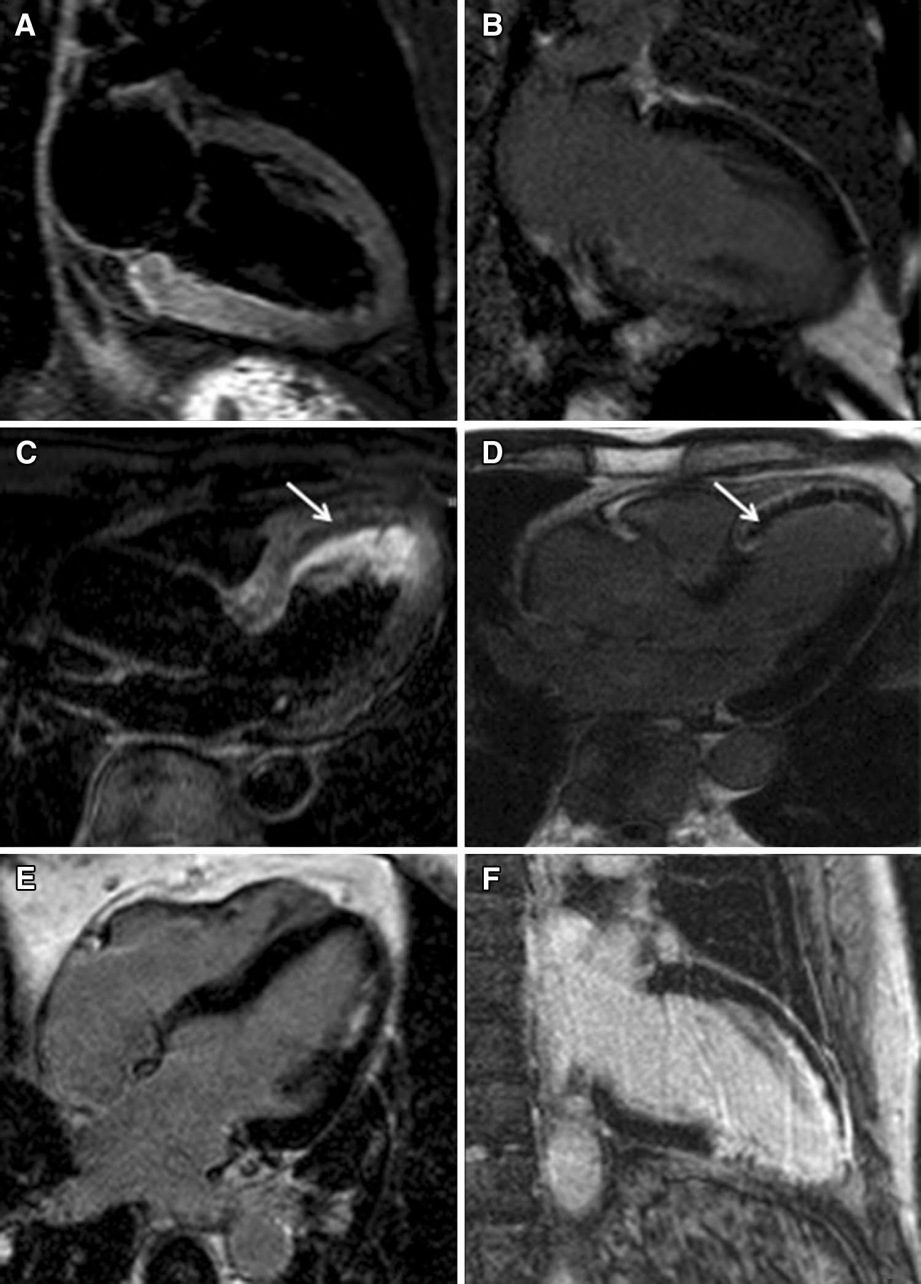
Myocardial infarction: overview. T2-weighted two chamber long axis view showing myocardial oedema in the basal to mid-cavity inferior wall (**a**) with concomitant subendocardial late gadolinium enhancement (LGE) in the post-contrast sequence (**b**) in a patient with acute subendocardial infarction in the proximal to mid right coronary artery territory. T2-weighted three chamber long axis view showing myocardial oedema in the mid-apical anteroseptum with evidence of a hypo-intense core (**c**, *white arrow*), consistent with intramyocardial haemorrhage, in a patient with transmural infarction in the distal left anterior descending territory (**d**) with persistence of microvascular obstruction (**d**, *white arrow*). Four chamber long axis post-contrast view showing subendocardial LGE of the mid-cavity anterolateral wall (**e**). Two chamber long axis post-contrast view showing transmural LGE of the mid-apical inferior wall (**f**)

Acute coronary occlusion determines cytogenic and vasogenic myocardial oedema, which is a rather non-specific response to an acute insult (ischemic, inflammatory, traumatic), and is characterized by an increase in water content [2]. CMR has the ability to distinguish fat, fluid and soft tissue by using different imaging protocols that exploit different intrinsic tissue properties [3, 4]. The linear correlation between myocardial water content and T2 transverse relaxation time in acute myocardial infarction was first described in the early 80s; T2-weighted sequences were subsequently developed and implemented in clinical practice to assess the presence of myocardial oedema, which appears as an area of increased signal intensity (bright). Pathophysiologic model of acute ischemia after coronary occlusion shows a rapid onset of myocardial oedema, which can be detected as early as 30 min from symptoms onset on T2-weighted images (Fig. 1a) [5]. The territory of distribution of the infarct-related artery is at risk of potentially irreversible damage (myocardium at risk) if reperfusion does not occur promptly [6]. When myocardial blood flow is promptly restored, the irreversibly damaged myocardial area is significantly smaller than the area at risk. T2-weighted sequences can detect the presence and extent of the myocardium at risk and derive the amount of myocardial salvage by subtracting the extent of scarred myocardium in the post-contrast sequences (late gadolinium enhancement, LGE) (Fig. 1b) from the area at risk on T2-weighted images. T2-weighted sequences for the assessment of the area at risk have been validated in animal studies versus the gold standard fluorescent microspheres, showing comparable findings [7]. The assessment of salvage myocardium is reproducible and has been validated against SPECT and angiographic studies [5]; moreover, while SPECT tracer needs to be given prior to revascularization, CMR provides the opportunity to assess salvage myocardium retrospectively, few days after the acute event, without interfering with the acute clinical management [8]. Identification of myocardial oedema in a patient with suspected acute coronary syndrome (ACS) not only confirms the diagnosis, but also helps in establishing the age of the myocardial infarction [9]. All patients presenting with ACS and those with high pre-test probability of CAD undergo coronary angiography in keeping with existing guidelines. However, in a non-negligible proportion of patients with ACS (approximately one-third), a “culprit” lesion is not identified by angiogram [10]; these cases are often attributed to undetectable coronary pathology, such as distal embolization, coronary spasm and myocardial bridging. According to the ischemic wave-front phenomenon, myocardial oedema extends from the subendocardium to the entire wall thickness of the territory supplied by the occluded vessel. Based on this pathophysiologic model, T2-weighted sequences can easily allow the detection of the culprit lesion, as an area of increased signal intensity along the distribution territory of a coronary artery (Fig. 1a). Monney et al. [11] found that myocardial infarction was identified in 13% of patients with ACS and unobstructed coronaries, as delineated by a localised increase in signal intensity on T2-weighted images in areas of transmural scar. Though persistence of myocardial oedema after an acute event varies according to different studies, infarct related oedema can persist from few weeks to few months after the event, so that it can also be detected once cardiac biomarkers have normalized [5]. As the abnormalities are transient, imaging these patients within 2 weeks from the event represents an optimal time window before the abnormalities resolve and become no longer detectable [12]. Not all patients in the NSTEMI-ACS population have evidence of CAD requiring revascularization; Raman et al. [13] showed that the presence of myocardium at risk on T2-weighted CMR sequences allows the identification of patients who will benefit from an early invasive management. In fact, the presence of myocardial oedema has been shown to increase the risk of adverse cardiovascular events, irrespective of revascularization [14]. Clinical evidence has shown that prompt myocardial revascularization reduces the infarcted area and the incidence of long-term adverse events. However, myocardial revascularization can itself also lead to myocardial damage, the so called “ischemia–reperfusion injury”, as shown by histology studies soon after revascularization. Prolonged ischemia-induced microvascular damage leads to absent distal myocardial flow (no-reflow phenomenon) on coronary angiography, which can be identified as either microvascular obstruction or intra-myocardial haemorrhage (IMH) on CMR. Both entities are a consequence of microvascular cell necrosis and present in the context of a large and late reperfused myocardial infarction [15]. Cardiovascular magnetic resonance soon after an acute ischemic event is able to detect the ischemia–reperfusion injury (Fig. 1c, d). The imaging equivalent of the angiographic and histologic no-reflow phenomenon is known as microvascular obstruction (MVO) and appears as a non-perfused, non-contrast gaining area within the infarcted myocardium (Fig. 1d); this can be detected after contrast administration and has typically low signal intensity (black) both in the early and late acquisition (5 and 15 min after contrast administration, respectively). As contrast agent needs time to diffuse into the non-perfused area, MVO extent also depends on time to image acquisition, appearing larger early after contrast administration and smaller when the images are acquired later as the contrast ultimately fills in these areas [16]. Extensive late MVO thus represents more severe microvascular damage [8]. Rochitte et al. [17] have shown that there is a time-course of MVO development, with onset within the first 3.5 h from the acute event, and subsequent expansion over the following 48 h. MVO increases up to three times during the first 2 days, reaching a steady-state between day 2 and 9, that represent the best time to quantify MVO [18]. By reflecting extensive microvascular damage, MVO has been shown to provide important prognostic insights. There is a linear relation between MVO extent and scar size, not only in the acute but also in the chronic setting, as the acute MVO is a predictor of transmural extent at 6 months [19]. MVO is a powerful predictor of adverse cardiovascular outcome, also independent of left ventricular ejection fraction (LVEF) [8, 19–22]. On a cohort of 438 reperfused STEMI patients undergoing CMR within 3 days from the acute event, in contrast to early MVO, only late MVO independently predicted adverse events [23]. De Waha et al. also showed that the ratio MVO/scar size is a more powerful predictor of adverse outcome then either considered alone [24]. Extravasation of erythrocytes into the myocardium as a consequence of disruption of capillaries and break-down of capillary barrier can be easily detected as a dark core area on T2-weighted images (Fig. 1c), due to the intrinsic paramagnetic properties of haemoglobin breakdown products, which shorten T2 relaxation time. Intramyocardial haemorrhage has been shown to be significantly related to infarct size and time to reperfusion, both in experimental models and in patients after percutaneous or surgical myocardial revascularization [15]. Out of 346 patients undergoing CMR after acute reperfused STEMI, Eitel et al. [25] found evidence of IMH in 35% of patients; in a multivariable model, infarct size, MVO extent and impaired LVEF were the strongest predictors of IMH. As expression of severe reperfusion injury, IMH has proved to have prognostic implications [25–27], demonstrating a strong unadjusted association with major adverse cardiovascular events [25, 27]. Intramyocardial haemorrhage is also a determinant of adverse left ventricular remodelling at 6 months follow-up; each myocardial segment showing IMH on T2-weighted images increases the risk of dilated left ventricular end-systolic volume by 50% [27].

## Complications of acute myocardial infarction

**Fig. 2:**
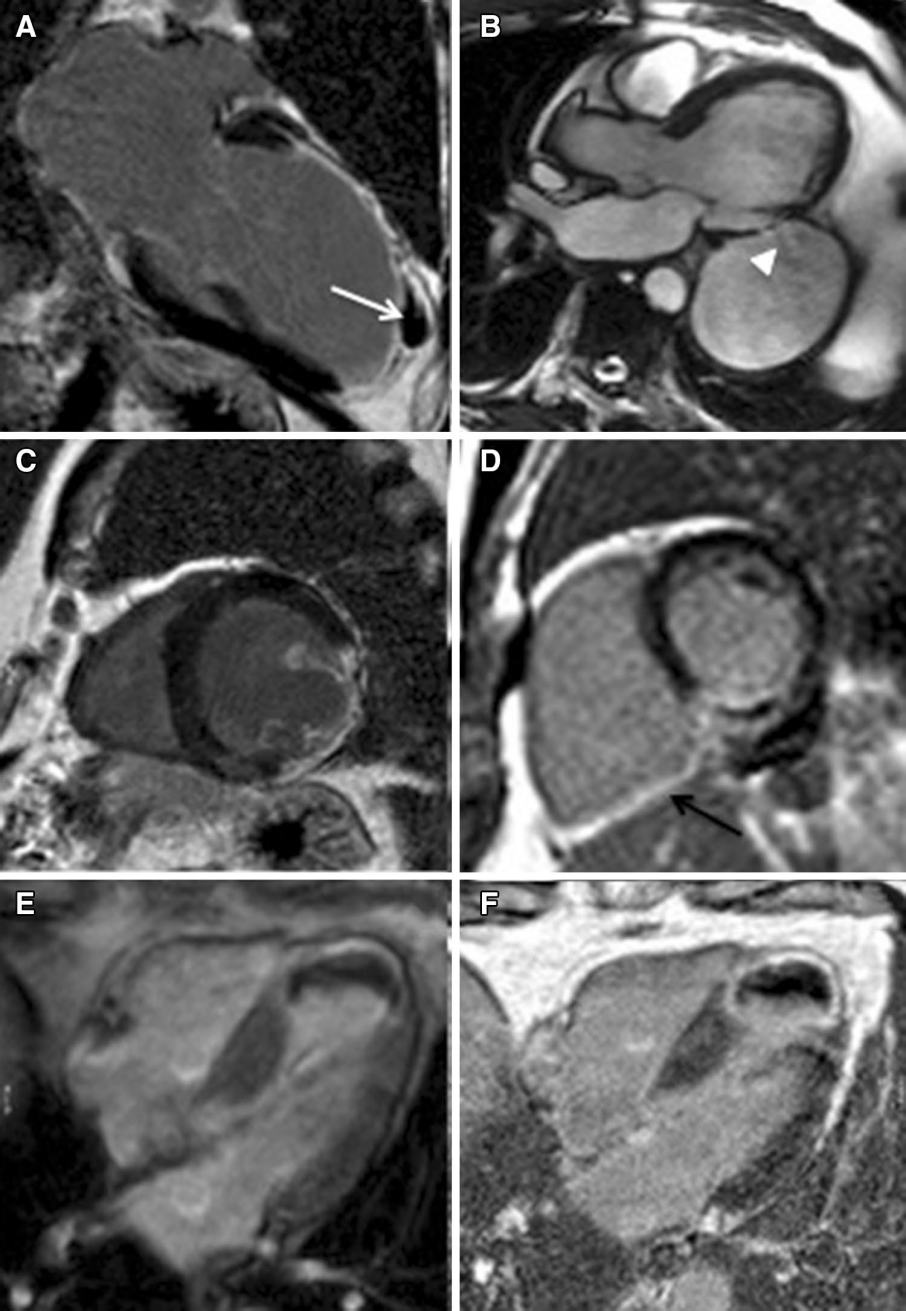
Complications of acute myocardial infarction. Two chamber long axis post-contrast sequence showing contained chronic rupture of the anterior wall (**a**, *white arrow*) in a patient with transmural myocardial infarction in the left anterior descending territory. Three chamber long axis cine sequence showing large pseudo-aneurysm of the mid-cavity inferolateral wall (**b**) with evidence of flow (**b**, *white arrow*-*head*) between a “tunnel-like” connection with the left ventricle in a patient with trasmural infarction in the left circumflex territory. Mid-cavity short-axis post-contrast sequence showing myocardial infarction of the papillary muscles (**c**). Mid-cavity short-axis post-contrast sequence showing myocardial infarction of the right ventricular inferior wall (**d**, *black arrow*) in a patient with transmural infarction in the basal inferior wall. Early (**e**) and late (**f**) four chamber long axis gadolinium enhancement sequences showing a large apical thrombus in a patient with transmural myocardial infarction in the distal left anterior descending territory (**f**)

With the onset of the reperfusion era, mechanical complications of acute MI, which are associated with reduced short- and long-term survival, have reduced to <1% [28] (Fig. 2). The most encountered mechanical complications include ventricular free wall (Fig. 2a) or septal rupture, papillary muscle infarction (Fig. 2c) or rupture with secondary acute mitral regurgitation (MR), ventricular aneurysm and pseudo-aneurysm (Fig. 2b). With its superior spatial resolution and feasibility soon after the acute event, CMR is a promising tool for the identification of early and late MI complications [20]. Post MI ventricular septal defect (VSD) is associated with 80–90% mortality within few months after acute event; not only is CMR useful to make the diagnosis, but also tissue characterization to look for fibrosis of VSD edges helps identifying the most appropriate time for surgery [20]. Myocardial tissue characterization by CMR also allows to distinguish a true aneurysm, which is characterised by a large neck and typically enhanced aneurysm wall, from pseudo-aneurysm, which usually has a small neck, with non-enhanced wall, and represents a contained rupture [20]. A lipomatous metaplasia seen as a high signal intensity area in the cine and post-contrast sequences, is also commonly seen in patients with old MI who underwent surgical myocardial revascularization [20]. The right ventricle (RV) is involved in a non-negligible proportion of acute MI, mainly involving the inferior wall (incidence 24–50%) [29], is haemodinamically relevant in up to 25–50% of cases and is usually associated with poorer prognosis [30, 31] (Fig. 2d). CMR has shown to detect RV involvement in 25% of patients with inferior MI, a percentage that is significantly superior to that detected by ECG and trans-thoracic echocardiogram (TTE). CMR studies have shown that RV myocardial infarction is usually associated with larger MI, lower LVEF and lower RV ejection fraction [30, 32]. LV thrombus is a frequent complication of ischemic heart disease, whose incidence increases with poor LVEF, greater scar size and ischemic aetiology [33] and whose diagnosis once relied on TTE. Given thrombus is avascular, it typically appears as a non-contrast gaining structure early (Fig. 2e) and late (Fig. 2f) after gadolinium administration on CMR; detection of thrombus by CMR has shown to be superior to TTE [34], also after contrast administration [35].

## Acute coronary syndromes with normal angiogram

**Fig. 3:**
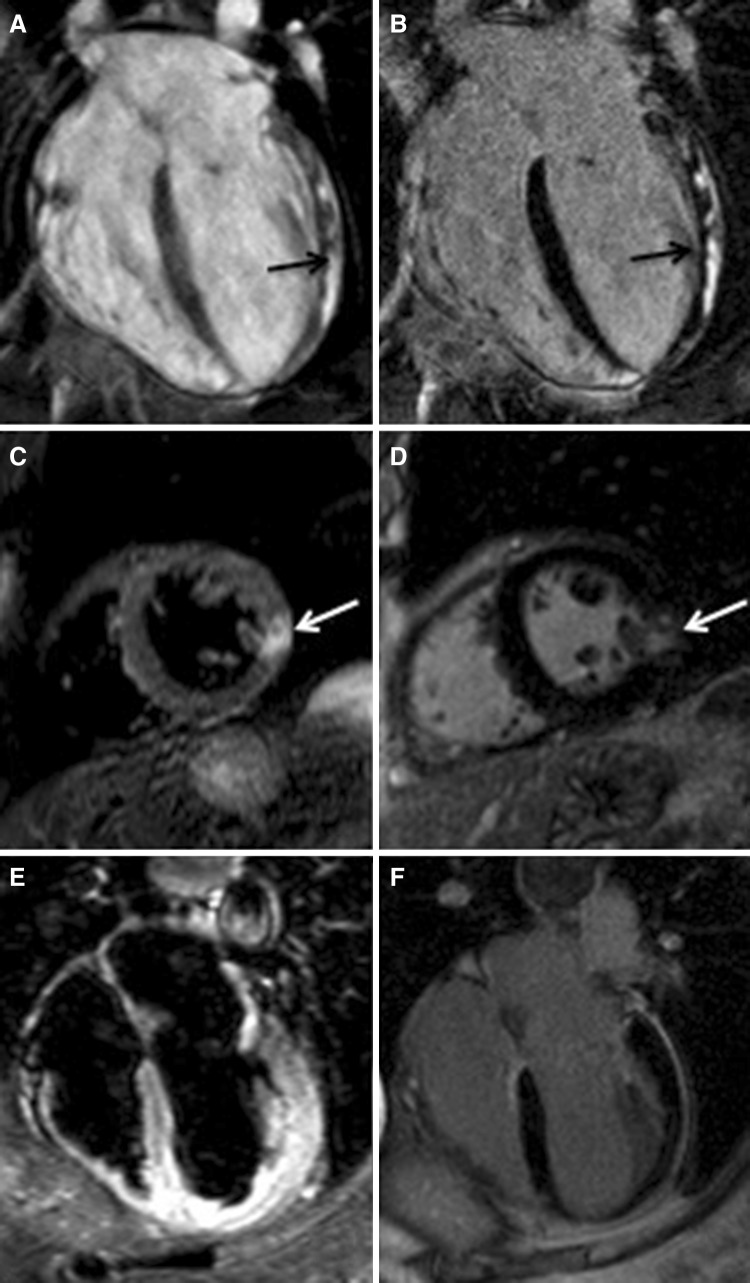
Acute coronary syndromes with normal angiogram. Early gadolinium enhancement sequences showing extensive epicardial enhancement (**a**, *black arrow*) and corresponding late gadolinium (LGE) on the post-contrast sequences (**b**, *black arrow*) in a patient with acute myocarditis. T2-weighted images showing focal discrete oedema in the mid-cavity inferolateral wall (**c**, *white arrow*) with corresponding LGE on post-contrast sequences (**d**, *white arrow*) in a patient with embolic myocardial infarction in the mid left circumflex territory. T2-weighted sequences showing acute myocardial oedema in the apical segments (**e**) with no evidence of late gadolinium enhancement (LGE) on the post-contrast sequences (**f**) in a patient with Tako-Tsubo cardiomyopathy

Up to a third of patients presenting with ACS shows normal arteries on coronary angiogram [12], making diagnosis and management challenging. Identifying a final diagnosis is important to guide patients’ management as it has implications both on medical therapy (secondary prevention if it was a confirmed ACS) and prognosis [12]. A recent meta-analysis on patients with myocardial infarction and unobstructed coronaries reported in-hospital and 12 months all-cause mortality of 0.9 and 4.7%, respectively [36]. Among the differentials, the commonest causes of ACS with normal angiogram are acute myocarditis, MI secondary to distal embolization, coronary spasm or spontaneous recanalization and Tako-Tsubo cardiomyopathy (TTC). CMR plays a pivotal role in the diagnosis of these entities, mainly based on its higher spatial resolution and superior tissue characterization properties [37, 38]. The unique selling point of CMR is indeed its ability to characterize myocardial tissue, based on the typical distribution pattern of the contrast agent, gadolinium: ischemic cardiomyopathy, following the ischemic wave-front, is typically characterized by late gadolinium enhancement (LGE) with a subendocardial to transmural distribution, while non-ischemic cardiomyopathy shows LGE with a mid-wall or epicardial distribution pattern, generally not located in the territory of distribution of a coronary artery [39]. A meta-analysis on more than 500 patients presenting with MI and unobstructed coronaries showed that one-third of patients had findings consistent with myocarditis [40] (Fig. 3a, b). Epicardial or mid-wall increased signal intensity on T2-weighted and early gadolinium enhancement sequences (Fig. 3a), with additional LGE in the post-contrast sequences (Fig. 3b), are the typical findings of acute myocarditis on CMR, which accurately resemble histologic Lake Louise criteria [41]. CMR is now recommended by European guidelines as first line imaging technique, prior to endomyocardial biopsy, in stable patients with suspected myocarditis [42]. Some studies suggest that myocarditis is a dynamic process which spreads from focal to disseminated myocardial involvement; CMR can timely follow-up this dynamic process [43]. The findings of LGE ischemic distribution pattern on CMR allows the identification of patients with “true” MI despite unobstructed coronaries on angiogram, such is the case of distal plaque embolization, coronary spasm or paradoxical embolization from a patent foramen ovale [38] (Fig. 3c, d). Tako-Tsubo cardiomyopathy, otherwise known as broken-heart syndrome or stress cardiomyopathy, accounts for up to 0.7–2.5% of all ACS, up to 12% in the female population [16] (Fig. 3e, f). TTC frequently presents as ACS-STEMI with evidence of unobstructed coronaries; it typically shows peculiar regional wall motion abnormality, involving the mid-distal segments of the heart. TTC is a reversible cardiomyopathy, which usually resolves within 6 months from the acute event, and can be detected on ventricular cine-angiogram and on TTE, mainly based on the typical regional wall motion abnormality. CMR provides an added value based on tissue characterization, showing a transient myocardial injury, characterised by the presence of myocardial oedema on T2-weighted images (Fig. 3e). Though it was once believed that in TTC LGE is absent, LGE has been found in a non-negligible proportion of cases, probably as a reflection of an underlying inflammatory response and transitory increase of extracellular space due to the myocardial oedema [44]. This is however reversible and no longer observed at follow-up in conjunction with the resolution of myocardial oedema/inflammation. The diagnostic value of CMR in patients with acute chest pain has been tested in the Emergency Department in the assessment of stable patients with chest pain, non-diagnostic electrocardiogram (ECG) and increased cardiac enzymes; in a study on 161 stable patients with acute chest pain, CMR showed high sensitivity and specificity in detecting ACS, being more sensitive and specific than the ECG [45].

## Chronic ischemic heart disease

Due to its ability to identify myocardial fibrosis, CMR has had its widest application in the assessment of chronic ischemic heart disease (IHD) [46, 47]. Detection of fibrosis is based on the analysis of the distribution of the contrast agent within the myocardium, 10–15 min after its administration. CMR uses gadolinium-chelate contrast agent, an extra-cellular agent, which is quickly washed out by normal myocardium, and accumulates in damaged myocardium with expanded extra-cellular space [39]. Whether the extra-cellular space is expanded because of myocardial cell rupture in the setting of acute myocardial infarction or because of collagen deposition in the setting of chronic myocardial scarring, gadolinium will accumulate and be detected on CMR. Myocardial fibrosis can be present as infarct, replacement or diffuse fibrosis; these represent a continuum of myocardial damage, rather than isolated entities, as shown by Beltrami et al. [48] who found that replacement and diffuse fibrosis account for 70% of all fibrotic tissue in end-stage IHD, while infarct fibrosis only accounts for 30%. The accuracy of CMR in assessing the ischemic scar has been validated in animal studies, which showed that the location, extent and shape of LGE on *ex-vivo* CMR were almost identical to the infarcted areas defined on histologic analysis [49]. CMR’s excellent contrast and spatial resolution allow the identification of even small amounts of infarct scar, below 1 g of mass [50, 51]. CMR has shown to be superior to single photon emission tomography (SPECT) in the assessment of myocardial scar, especially in cases of small infarct and infarct in a non-anterior location [52]. Infarct healing is a well-known process, that can easily be detected and followed-up by CMR. A study on 58 reperfused STEMI patients showed that infarct size reduces 4 months after the acute event, but is relatively unchanged at 1 year follow-up [53]; in the acute phase infarct volume is influenced by hyperaemia, oedema and inflammation, which might explain the overestimation of infarct size early after the acute event [54]. However, infarct size at baseline has proved to be the strongest predictor of adverse long-term LV remodelling [53], which continues for up to a year after the acute event, also involving the remote non-infarcted myocardium. There is a strong linear relation between scar size, LV end-systolic (LVESV) and end-diastolic volumes (LVEDV) and LVEF. Scar size is the strongest predictor of LVEF, independent of scar location and transmurality [55]. A study on 90 reperfused anterior STEMI undergoing CMR early (3–5 days) and 90 days from the acute event showed that baseline infarct size, infarct heterogeneity and myocardial salvage are significantly associated with 90-day LVEF [56].

## Myocardial viability

**Fig. 4:**
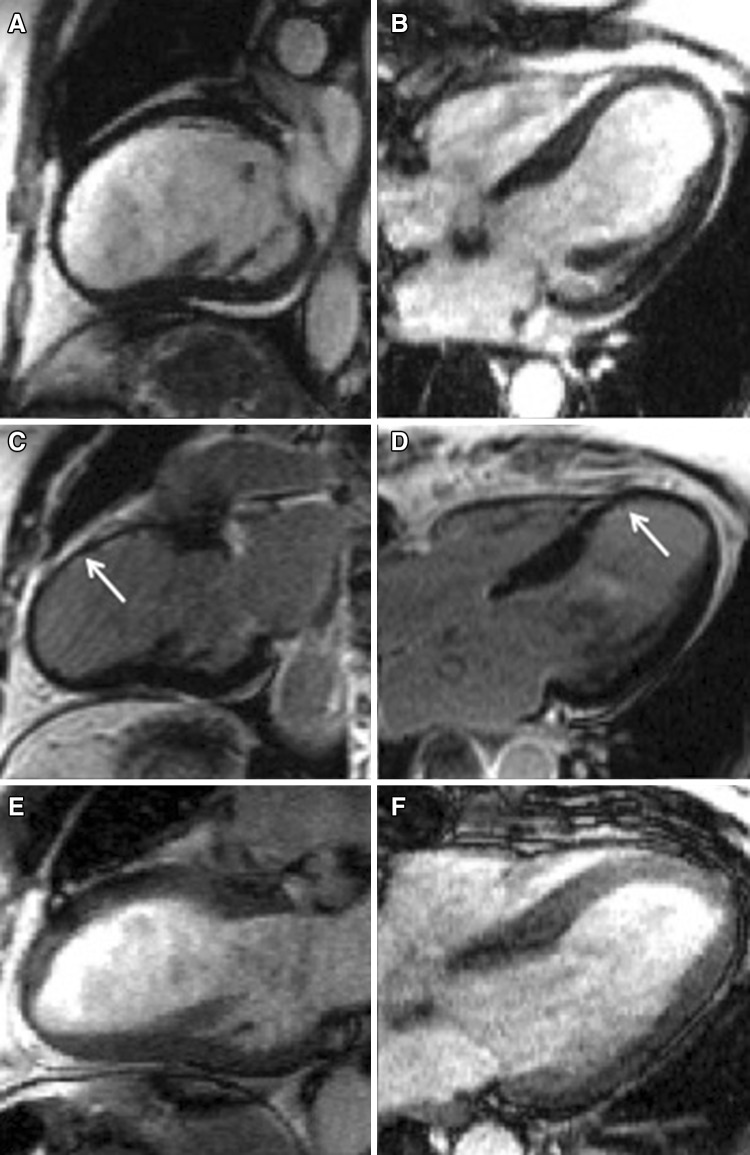
Myocardial viability and left ventricular (LV) recovery after revascularization. Long-axis two (**a**) and four chamber (**b**) cine sequences showing marked thinning of the LV anterior and anteroseptal walls in a patient with severe disease of the mid-distal left anterior descending (LAD); post-contrast sequences showing viable myocardium in the mid-distal LAD territory as late gadolinium enhancement (LGE) is limited to the subendocardium (**c, d**, *white arrow*). Long-axis two (**e**) and four chamber (**f**) cine sequences showing recovery of LV wall thickness 3 months after revascularization of the LAD

Transient ischemia impairs LV function, acutely and chronically, if repeated episodes occur; acute ischemic LV impairment is known as myocardial stunning, while chronic ischemic impairment is known as myocardial hibernation [57]. However, ischemic LV dysfunction is not necessarily an irreversible process, and LV function can improve after revascularization [58]. Myocardial viability is a reflection of impaired ischemia-induced contractility at rest that recovers after revascularization. The assessment of myocardial viability is the cornerstone to guide clinical treatment, as it has been shown that complete revascularization of viable myocardium reduces long-term adverse events, as compared to medical therapy [59]. Myocardial viability was once defined on TTE and PET, by means of LV wall thickness, wall motion abnormality on low-dose dobutamine echocardiography [57] and reduced metabolism. Low-dose dobutamine stress CMR has good specificity (83%) and moderate sensitivity (74%), not dissimilar from those of stress dobutamine echocardiography; a “bi-phasic response” is highly predictive of functional recovery [60]. The concept of myocardial viability was expanded by the implementation of scar analysis on CMR [61], which has shown better sensitivity, specificity and accuracy than SPECT in the prediction of myocardial viability [62]. Extent of LGE is significantly associated with myocardial viability and functional recovery. Kim et al. [63] assessed LGE extent and myocardial contractility before and after revascularization in 50 patients presenting with impaired LV systolic function, showing that improvement in LV contractility decreases with the increase in scar transmurality: 78% of LV dysfunctional segments with no LGE improved in function, compared to only 2% of those with evidence of >75% LGE. A study on patients undergoing CMR before, early (6 days) and late (6 months) after surgical revascularization, showed that LGE transmurality strongly related to recovery in regional function at 6 months [64]. Using a 50% trasmural viability cut-off, 10 viable + normal segments predicted ≥3% improvement in LVEF, with a sensitivity of 95% and specificity of 75% [65]. Different studies found that irreversibly damaged myocardium was characterised by reduced wall thickness, so that reduced end-diastolic wall thickness was predictive of no myocardial recovery after revascularization [66]. Though regional LV wall thinning has been long thought to represent chronic transmural scar, Shah et al. [67] showed that among patients with CAD found to have LV wall thinning (≤5.5 mm at end-diastole) 18% had only a small scar burden (≤50% of total extent), which was associated with improved contractility and resolution of wall thinning after revascularization (Fig. 4). The assessment of scar in IHD has important prognostic implications. First, it predicts late myocardial recovery soon after the acute ischemic event; LGE volume after acute STEMI proved to be the strongest predictor of late LV dysfunction, over and above infarct transmurality, MVO and myocardial salvage, with a hazard ratio of 6.1 for adverse events when LGE extent was ≥23% [68]. The association of greater infarct size and impaired LVEF predicts even poorer outcome [69]. Infarct size, LVEF and LVESV on CMR predict future cardiac events early (1 week) after acute STEMI [70]. Among patients with clinically suspected CAD but with no history of MI, evidence of LGE on CMR is associated with worse outcome, and remains the strongest predictor of adverse events, even after adjustment for significant CAD on angiogram, LVEF and wall motion abnormality [71, 72].

## Risk stratification

The assessment of infarct scar is important also to risk stratify patients according to their risk of arrhythmic events, as it is well known that myocardial scar represents the substrate for re-entrant arrhythmias [73]. Different studies on patients undergoing CMR prior to ICD implantation have shown that scar extent is stronger than LVEF in predicting arrhythmic events (sudden death, ICD discharge, ventricular arrhythmias) and inducibility at electrophysiologic study (EPS) [74–76] and remains the strongest predictor, also in patients with preserved ejection fraction [77]. Scar analysis on CMR is based on different signal intensity of the infarcted area as compared to an area of normal, remote myocardium. Most studies have defined the cut-off for abnormal signal intensity at 5 SD above that of normal myocardium to identify the core infarct and between 2 and 3 SD above normal myocardium to identify the peri-infarct zone, though the full width at half maximum technique has shown to be the most accurate and reproducible, regardless of the underlying myocardial disease [78]. Detailed semi-automated scar analysis allowed the identification of an even higher association between the peri-infarct zone and the arrhythmic risk. It is now believed that tissue heterogeneity is the strongest predictor of EPS inducibility and freedom from recurrence after ventricular tachycardia ablation [79, 80]. CMR is thus a valuable tool also in the assessment of patients prior to ICD implantation, and there is recent evidence that scar extent on CMR predicts response to cardiac resynchronization therapy (CRT) [81, 82]; in a study on 47 IHD patients undergoing CRT, CMR showed that response rate to CRT was higher in patients with higher LVEF, smaller scar and lower number of LV segments with >51% scar transmurality [82]. Finally, primary prevention ICD implantation is based on LVEF [83]. CMR is the gold standard for the assessment of LVEF as it is free from any geometric assumption [84, 85]: based on full 3D coverage of the heart, by contouring the endo- and epicardial borders, CMR provides 3D volumetric assessment of ventricular volumes and function.

## Stress perfusion CMR

**Fig. 5:**
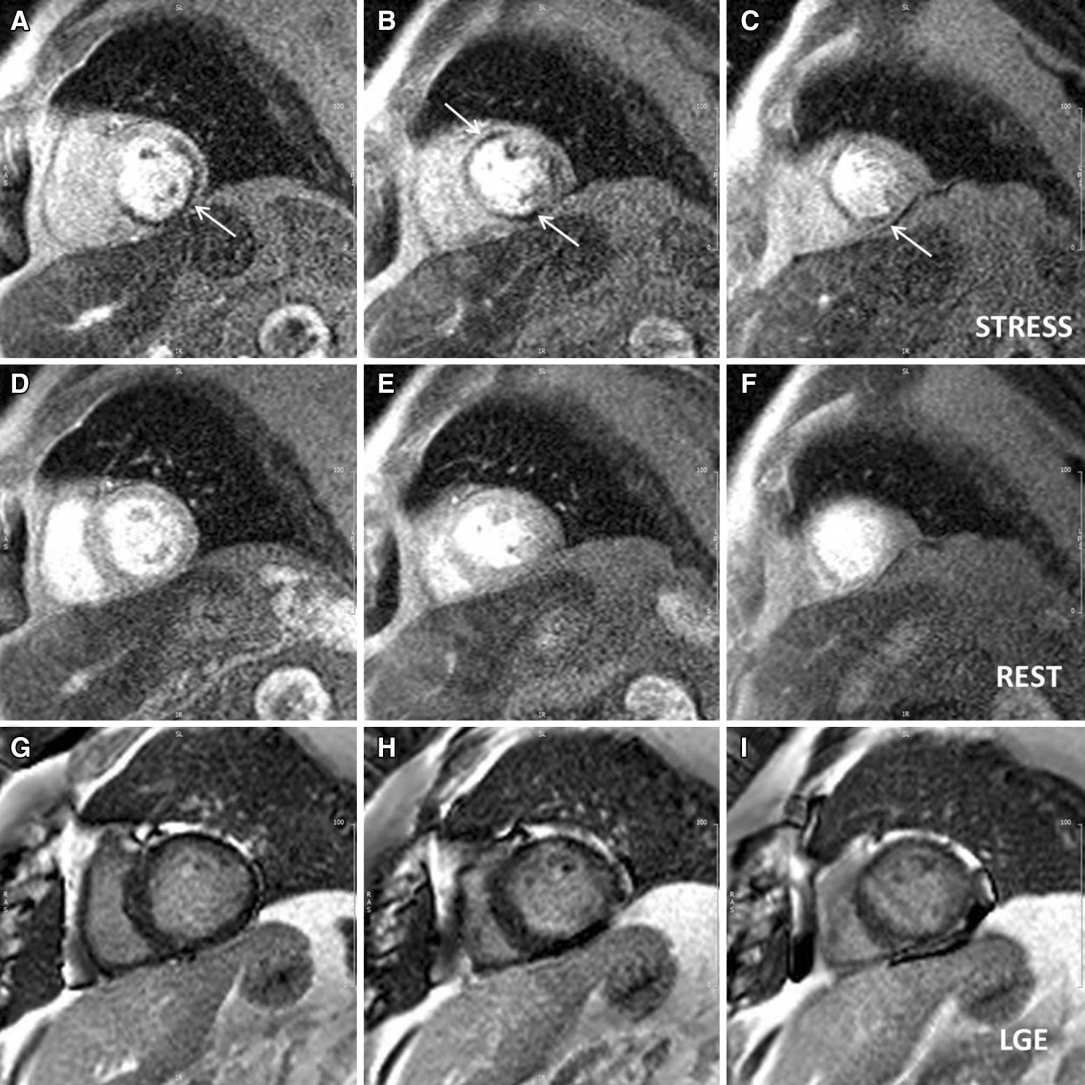
Stress perfusion CMR. Three slice (base, mid and apex—**a, b** and **c**, respectively) short axis stress perfusion sequences acquired at peak adenosine infusion showing two separate areas of hypoperfusion, in the proximal to distal inferior (**a**–**c**, *white arrow*) and mid-cavity anterior walls (**b**, *white arrow*), with a normal rest perfusion (**d**–**f**) and only a discrete area of subendocardial late gadolinium enhancement in the mid-cavity anterior wall (**h**) on post-contrast images (**g**–**i**). Overall, these findings are consistent with inducible myocardial ischemia in all right coronary artery territory with ischemia superimposed to the infarcted area in the mid left anterior descending territory

Stress CMR has been recently recognised as a reliable technique to diagnose myocardial ischemia in the setting of CAD. It is based on the assessment of myocardial perfusion during pharmacological stress testing with coronary vasodilators (Fig. 5), and on the detection of inducible wall motion abnormalities during high-dose dobutamine infusion (HDD-CMR). The most used vasodilator is adenosine, mainly because of its short-life and limited side effects; adenosine increases coronary artery vasodilatation in normal coronaries, but it does not increase blood flow downstream to stenotic arteries as the arteriolar bed is already maximally dilated (so-called “coronary steal” phenomenon); this allows the identification of areas of hypoperfused myocardium distal to a significant stenosis [86], which appear as a low signal intensity (dark) area on stress perfusion images (Fig. 5a–c). Stress perfusion CMR showed good diagnostic performances in several studies [87]. In a meta-analysis of 12 studies (761 patients) using fractional flow reserve (FFR) as a reference standard, perfusion CMR had a sensitivity of 89.1% and specificity of 84.9% on a patient basis as well as a sensitivity of 87.7% and specificity of 88.6% on a coronary territory basis [88]. Nandalur et al. showed that perfusion CMR has a sensitivity of 91% and a specificity of 81% in a per-patient analysis, and of 84 and 85% respectively for the identification of the ischemic segments [89]. Recently, a large randomized trial has shown that perfusion CMR has better sensitivity and negative predictive values compared to SPECT, and that it offers an accurate assessment of single-vessel and multi-vessel coronary disease, irrespective of the cut-off used for defining clinically significant coronary artery stenosis [90].

Perfusion CMR assessment of coronary flow reserve has also been compared with PET, and there was an excellent correlation between the two modalities [91]. Dobutamine is a sympatomimetic amine with positive inotropic and chronotropic effects, that mimics the physiological effect of physical exercising, inducing increased oxygen demand [86]. When administered at low-dose it usually determines the recruitment of the contractile reserve of hibernating myocardial segments (dysfunctional but viable myocardium at rest), but when given at high-dose it induces ischemia in territories with significant coronary artery stenosis, which is expressed as the onset of wall motion abnormalities, easy to recognize on CMR cine images. HDD-CMR has been shown to be superior to dobutamine stress echo (DSE) with significantly higher diagnostic accuracy, probably due to superior image quality allowing a better assessment of regional wall motion abnormalities [92]. However “the visual assessment-based” interpretation of wall motion abnormalities can lead to different results. Recently, the introduction of the feature tracking software (FT), allowing the assessment of myocardial strain from cine images, without the need for additional sequences, has improved this limitation. Schneeweis et al. showed that FT based analysis of circumferential strain during HDD-CMR was feasible and helped differentiating between normal myocardium and segments supplied by a stenotic coronary artery, suggesting that the quantitative assessment of myocardial strain with FT may improve the diagnostic accuracy of HDD-CMR for detection of ischemia [93].

## Prognosis

Many studies have been published regarding the assessment of prognosis with stress-CMR [94, 95]. Sozzi et al., showed that among 326 consecutive patients with normal adenosine stress perfusion CMR the event rate was low and prognosis excellent over a 5.5-year period [96]. Kelle et al. found that, in a large cohort of patients with negative dobutamine stress, the annual cardiac event rate was 1.1% while the hazard ratio associated with a positive test was 3.3 [97]. In a large meta-analysis of 19 studies, involving a total of 11,636 patients with a mean follow up of 32 months, Lipinski et al. found that a negative stress CMR was associated with very low-risk of cardiovascular death and myocardial infarction, suggesting that stress CMR may help in the risk stratification of patients with known or suspected CAD [98]. Based on those data, current clinical guidelines for myocardial revascularization, suggest the use of stress CMR in the management of patients with intermediate risk of CAD and stable symptoms (class IA) [99].

The 5-year follow up of the CE-MARC study demonstrated that compared to SPECT, CMR is the strongest predictor or risk for MACE, independent of cardiovascular risk factors, angiographic result, or initial patient’s treatment [100].

## Indications of CMR in ischemic heart disease: international guidelines

According to international guidelines, CMR plays a role in the assessment of patients with ischemic heart disease. 2013 ESC guidelines on stable angina recommended imaging stress testing (IB) for risk stratification in patients with known stable CAD and a deterioration of symptoms as a guide to clinical decision making and in the assessment of patients with intermediate pre-test probability (15–85%) of CAD [101]. Imaging stress test was also recommended in symptomatic patients with prior myocardial revascularization or to assess functional severity of intermediate lesions on angiography (IIaB). Stress testing with CMR is also recommended before or after discharge to assess residual myocardial ischemia and viability in STEMI patients (IA) [1]. More recently, stress CMR has been given a class IA indication by the new European Society of Cardiology guidelines on myocardial revascularization [99] to assess suspected CAD in patients with intermediate pre-test probability.

## Limitations

Though CMR is increasingly used, its availability is still limited in certain centres. CMR can definitely be performed safely also in the acute setting, but the patient needs to be haemodinamically stable. There are few contraindications to CMR. Patients with non-MR conditional devices (intracranial clips, neuro-stimulator, metallic objects in the eye) should not be offered a CMR; recent advances in technology do provide MR-conditional cardiac devices (pace-maker and ICD), that allow CMR scanning, though under strict medical monitoring. Gadolinium-chelate contrast agent is safer than iodine contrast agents, but should be avoided in severe renal dysfunction (eGFR < 30 ml/min/1.73 m^2^), as it increases the risk of nephrogenic systemic fibrosis, a potentially fatal condition. Claustrophobia has long been thought to be an absolute contraindication to CMR; however, performing CMR with the patient lying prone, providing an angulated mirror in the CMR bore to allow the patients to look outside the scanner and inviting a relative to sit at the end of scanner should help reduce these cases to a negligible percentage.

## Conclusion

CMR is a well-established imaging tool, which allows a comprehensive, multi-parametric cardiac assessment in a 40-min one-stop-shop technique. The use of CMR in ischemic heart disease has rapidly spread given its superior diagnostic properties and important prognostic implications.
